# Influence of Bone Conduction Hearing Implantation on Health-Related Quality of Life for Patients with Chronic Otitis Media

**DOI:** 10.3390/jcm11185449

**Published:** 2022-09-16

**Authors:** Aaran T. Lewis, Viktor Gergely

**Affiliations:** 1 School of Public Health and Community Medicine, University of Gothenburg, 40530 Gothenburg, Sweden; 2 Cochlear Limited, 43533 Mölnlycke, Sweden

**Keywords:** chronic otitis media, cholesteatoma, hearing loss, hearing rehabilitation, quality of life, bone conduction implant, utility gain

## Abstract

Background: Chronic otitis media is a major public health burden that can result in a disabling hearing loss. Bone conduction hearing implants are an accepted form of hearing rehabilitation in these patients, but evidence supporting their usage typically comes from studies investigating mixed indications. The objective of our study was to examine how these devices impact health-related quality of life and hearing-disability in adult patients suffering from chronic otitis media. Methods: Health Utilities Index-mark III (*n* = 10) and Speech Spatial and Qualities of Hearing-49 data (*n* = 6) were extracted for adult patients with chronic otitis media from an international hearing implant registry. Data were compared at baseline and at 12-month post-implantation with a bone conduction hearing implant. Results: Patients demonstrated a clinically relevant mean utility gain of 0.145 following implantation and clinically relevant mean improvement in global speech spatial and qualities of hearing score following implantation. Conclusions: Bone conduction implantation was found to improve hearing and health-related quality of life and reduce hearing disability in a small cohort of patients with chronic otitis media. These data highlight the importance of providing appropriate hearing rehabilitation for individuals with chronic otitis media.

## 1. Introduction

Chronic otitis media (COM) is an umbrella term that describes recurrent infection of the middle ear space and/or mastoid air cells, often leading to tympanic membrane perforation [1]. The disease usually develops from an acute otitis media during childhood and can develop with or without cholesteatoma [2]. Frequent symptoms associated with the disease include hearing loss, otorrhea, otalgia, and vertigo. The global prevalence of COM is estimated to be between 65 and 330 million people [3] and half of these individuals are projected to suffer from a disabling hearing loss, making a considerable contribution towards the global burden of hearing loss [3]. Hearing loss is a debilitating condition associated with several secondary complications, including learning difficulties, social isolation, depression, and dementia. This is a significant public health issue since untreated hearing loss negatively impacts cognition, physical health, mental wellbeing, and learning [4,5]. Hearing loss also disrupts social interaction and communication and degrades mental well-being [6,7,8]. Furthermore, the stigma associated with hearing loss can result in social isolation and increases the risk of developing psychological disorders [9]. The disease has also been shown to negatively impact a patient’s health-related quality of life [10], but there is need for indication-specific data to drive the accurate assessment of interventional benefits.

No current evidence-based approach to hearing rehabilitation has gained clinical consensus, but commonly performed middle ear surgeries do not always lead to satisfactory hearing outcomes [11] and bone conduction hearing devices could be an effective alternative when rehabilitating hearing in these patients [12]. Bone conduction hearing devices treat hearing loss by stimulating the cochlea using vibrations that are transmitted from the device through the skull. Bone conduction systems consist of an externally worn sound processor that is coupled to a titanium implant placed in the skull bone [13]. The external sound processor collects sounds from the environment and the system converts these to mechanical vibrations. In the case of percutaneous systems, these vibrations are sent to the cochlea via an abutment, which breaches the skin but provides direct stimulation. Transcutaneous systems send sound vibrations across intact skin, which provides a more aesthetically appealing outcome but results in some transmission losses [13]. These devices are indicated for use in patients with conductive hearing loss, mixed hearing loss, or single sided deafness that do not, or cannot, benefit from acoustic hearing aids. In patients with COM, a key advantage of these devices is that the ear canal is not occluded, minimizing moisture accumulation and skin irritation [14]. Therefore, bone conduction devices are widely considered for patients with persistent otorrhea, otitis externa and patients that are unable to wear conventional air conduction hearing aids. High levels of satisfaction in relation to sound amplification and speech perception when using bone conduction hearing devices have been reported in patients with conductive or mixed hearing loss [15]. However, there is considerable uncertainty surrounding how these interventions impact health-related quality of life (HRQoL) in patients with COM, since utility values for hearing device-assisted health states are lacking [16].

Alongside traditional clinical outcome measures, there has been an increasing need to complement these with additional measures that can capture how interventions impact patients’ HRQoL. Two widely used instruments are the Health Utilities Index Mark 3 (HUI-3) [17] and the Speech, Spatial, and Qualities of Hearing Scale (SSQ) [18]. The HUI-3 has been established as a sensitive measure to demonstrate the impact of a broad range of medical treatments covering 8-domains of health: vision, hearing, speech, ambulation, dexterity, emotion, cognition, and pain [19]. HUI-3 has been shown to be sensitive to changes in hearing-related quality of life and is often the recommended instrument for capturing these changes in studies evaluating hearing interventions [20,21]. The original SSQ consists of 49 questions: 14 scored items on speech-hearing, 17 on spatial-hearing, and 18 on other functions and qualities of hearing [18]. The SSQ is a widely used tool in routine clinical practice and hearing research and has been reported to be sensitive to changes in hearing function following treatment with a variety of hearing therapies, including implantable and non-implantable hearing devices [22,23].

In this study, we use HUI-3 and SSQ data collected as part of a voluntary registry study to determine the influence that bone conduction implants have on health-related quality of life in patients with COM, with or without cholesteatoma. Health-related quality of life data collected via HUI-3 will inform optimal care in this patient group and is a step towards reducing the global burden of hearing loss. These data can also be used to generate quality-adjusted life years, which can be used to inform cost-effectiveness models, permitting comparisons capable of supporting value-based healthcare.

## 2. Materials and Methods

### 2.1. Data Collection and Study Population

Data were collected as part of the study “Observation of Benefits for Patients Implanted with a Hearing Implant of the Company Cochlear (IROS)” (ClinicalTrials.gov Identifier: NCT02004353). IROS is a prospective, repeated measures, longitudinal study with intra-subject controls that uses subjective evaluation tools, including the HUI-3 and SSQ, to assess patient-related benefits following the treatment of permanent hearing loss with implantable hearing devices, including bone conduction hearing aids. All sites were required to obtain ethics committee approval prior to recruitment and no applications for ethical approval were rejected.

The registry contained baseline demographic and health outcomes data for 348 patients implanted with a bone conduction device and 12-month follow-up data, post implantation, for 76 patients implanted with a bone conduction device. Of these, 44/76 patients received a diagnosis of COM (*n* = 26) or cholesteatoma (*n* = 18). For HUI-3, baseline and follow-up data were available for 10 patients. For SSQ, baseline and follow-up data were also available for six of these patients with complete HUI-3 data. All patients were implanted unilaterally. Three patients received a percutaneous Baha^®^ Connect system and the seven remaining patients received the transcutaneous Baha Attract System.

### 2.2. Hearing Disability and Health Related Quality of Life

Patient-reported outcomes were collected at baseline and at 12-month follow-up after implantation with the bone conduction implant. Two questionnaires were used to collect information regarding HRQoL and hearing disability, respectively: HUI-3 [17] and the SSQ [18]. HUI-3 evaluates 8 HRQoL dimensions: vision, hearing, speech, mobility, dexterity, self-care, emotion, and cognition. The instrument also provides a comprehensive health state attribute. SSQ measures the self-reported auditory disability in everyday life across three subdomains (speech, spatial, and qualities of hearing). Single-attribute scores and overall scores at baseline and 12-month follow-up for both instruments were calculated for these patients and tested for clinical relevance and statistical significance.

### 2.3. Statistical Analysis

Data were analyzed in IBM SPSS, version 17. Owing to the small sample size, paired *t*-tests were used to determine any difference in mean utility score between pre- and post-implantation, and outcomes are reported as means since HUI-3 and SSQ data are routinely reported using mean values to facilitate comparison across studies.

Descriptive statistics are presented as means with standard deviations and one-tailed *p*-values are reported since it is expected that hearing implants will improve hearing in hearing impaired individuals. Effect sizes are calculated and presented according to Cohen’s D (d), where 0.2, 0.5, and 0.8 represent small, medium, and large effect sizes, respectively [24]. Unlike significance testing, the effect size is independent of sample size and can be used to determine the magnitude of an effect, even when the sample size is small.

A change in overall HUI-3 HRQoL score of 0.03 is considered clinically important and changes in single attribute scores of at least 0.03–0.05 have been shown to be clinically relevant [17,25]. Regarding SSQ, differences in rating change of 1.0 are considered clinically important [18].

## 3. Results

### 3.1. Demographics

Ten patients with COM, with or without cholesteatoma, completed the HUI-3 pre-implantation and ten of these individuals completed the questionnaire at 12-month follow-up, post-implantation. Regarding SSQ, six patients with COM, with or without cholesteatoma, completed the SSQ pre-implantation and six of these completed the questionnaire at 12-month follow-up, post-implantation. Demographic information and pre-implantation bone conduction hearing thresholds for each group used in the analysis are presented in Table 1.

The HUI-3 dataset consisted of baseline and 12-month follow-up data from ten patients. The SSQ dataset consisted of baseline and 12-month follow-up data from six of these patients. There were no missing questionnaire responses in the HUI-3 data. There were six questionnaire responses missing at random throughout the SSQ, representing less than 1% of the dataset, and these were replaced with the mean score of the subscale in which data were missing.

### 3.2. Health Utilities Index Mark 3

**Figure 1:**
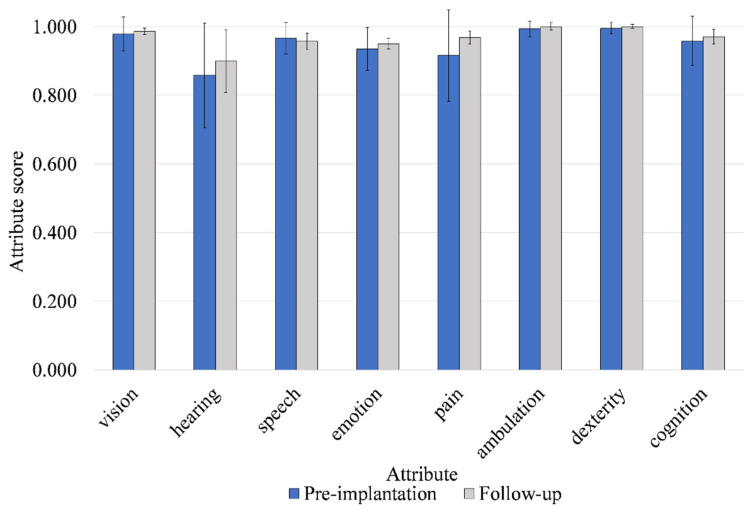
Mean HUI-3 single attribute scores pre-implantation and at 12-month follow-up (*n* = 10). Error bars represent the standard deviation of the mean. The improvement in hearing and pain attribute was clinically relevant between pre-implantation and 12-month follow-up.

**Figure 2:**
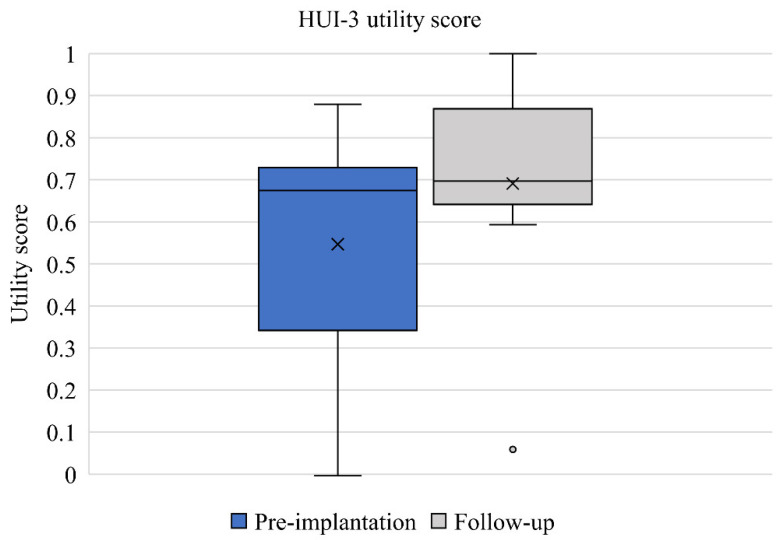
Distribution of HUI-3 utility scores pre-implantation and at 12-month follow-up (*n* = 10). Median values are represented by lines and means by crosses.

HUI-3 scores pre-implantation and at 12-month follow up, post-implantation (*n* = 10), are presented in Table 2. Mean attribute scores for all attributes other than speech improved between baseline and follow-up (Figure 1). Mean utility score also improved from 0.546 to 0.691 between pre-implantation and follow-up (Figure 2). The mean score improvements in hearing and pain attribute were clinically relevant but did not reach statistical significance. The improvement in utility score of 0.145 did not reach statistical significance but was clinically relevant and had a medium effect size (d = 0.538).

### 3.3. Speech, Spatial and Qualities of Hearing Scale-49

**Figure 3:**
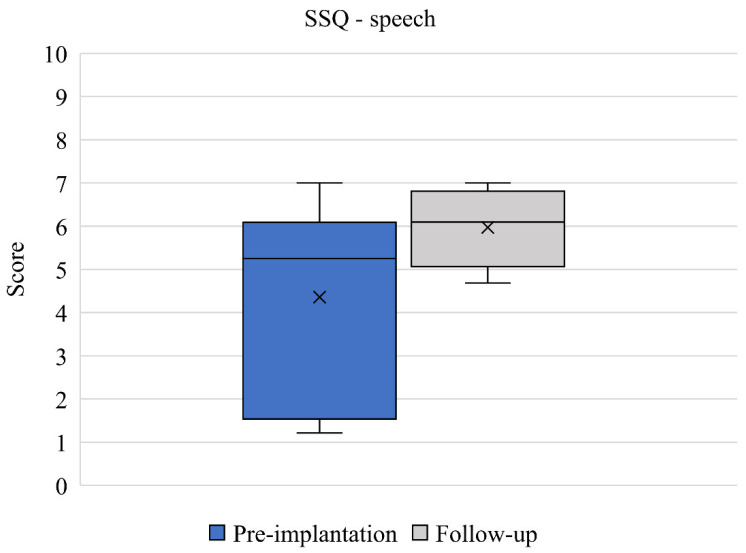
SSQ speech domain scores pre-implantation and at 12-month follow-up (*n* = 6). Median values are represented by lines and means by crosses.

**Figure 4:**
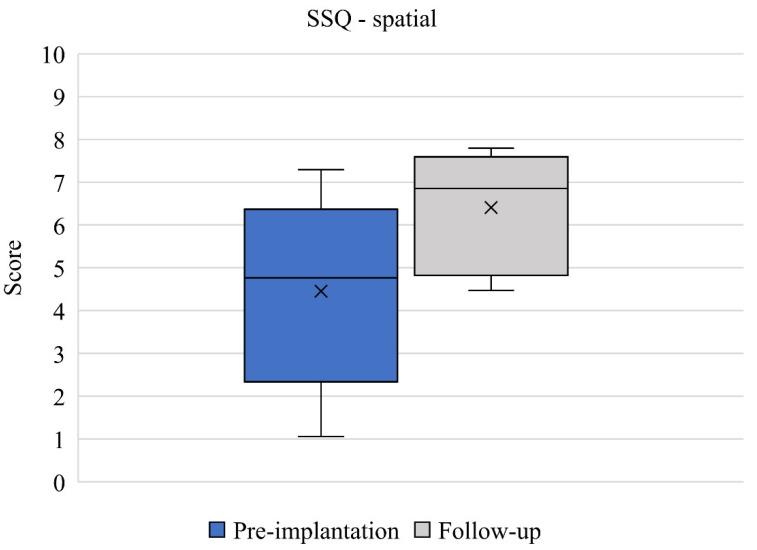
SSQ spatial domain scores pre-implantation and at 12-month follow-up (*n* = 6). Median values are represented by lines and means by crosses.

**Figure 5:**
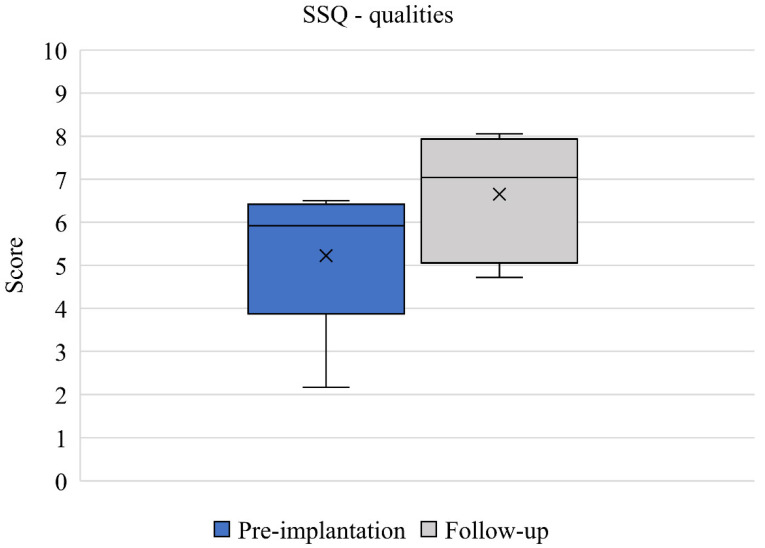
SSQ qualities domain scores pre-implantation and at 12-month follow-up (*n* = 6). Median values are represented by lines and means by crosses.

**Figure 6:**
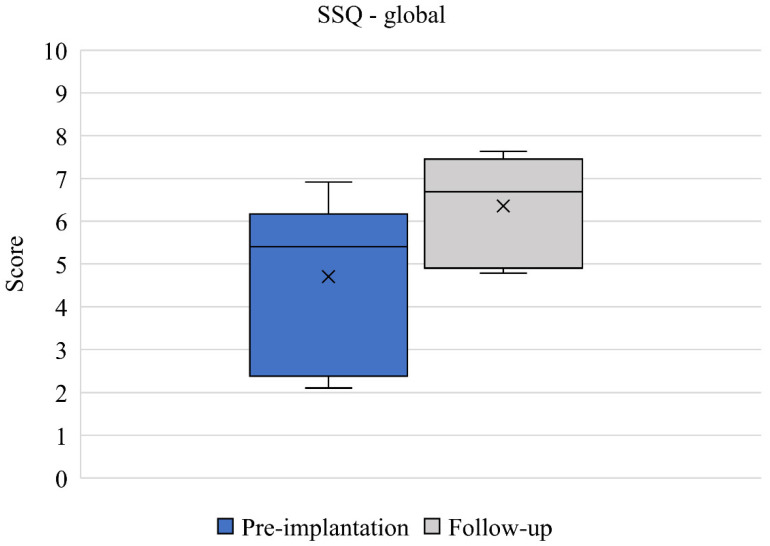
Global SSQ scores pre-implantation and at 12-month follow-up (*n* = 6). Median values are represented by lines and means by crosses.

SSQ results between pre-implantation and 12-month follow-up (*n* = 6) are presented. Mean speech domain scores improved from 4.36 (2.36) to 5.97 (0.97) between baseline and 12-month follow up (Figure 3). This change was clinically relevant but did not quite reach statistical significance (*p* = 0.052) and the effect size was large (d = 0.892). Mean spatial domain scores improved from 4.45 (2.33) to 6.41 (1.42) between baseline and 12-month follow up (Figure 4). This change was both clinically relevant and statistically significant (*p* = 0.027) and the effect size was large (d = 1.012). Mean qualities of hearing domain scores improved from 5.22 (1.67) to 6.63 (1.40) between baseline and 12-month follow up (Figure 5). This change was clinically relevant and statistically significant (*p* = 0.034) and the effect size was large (d = 0.921). Finally, mean global SSQ scores improved from 4.70 (1.98) to 6.35 (1.26) between baseline and 12-month follow up (Figure 6). This change was clinically relevant, statistically significant (*p* = 0.033), and the effect size was large (d = 0.994).

## 4. Discussion

Interventional studies with bone conduction devices are routinely conducted on patients with mixed indications, making it difficult to draw conclusions regarding how implantation may influence patient outcomes in specific disease indications, such as COM. Furthermore, it is important to measure functions which may influence HRQoL and hearing handicap via self-reporting, since many of these functions are difficult to measure objectively in a laboratory setting on clinical populations. Self-reported assessments provide valuable insights into the health deficits faced by individuals in the real-world. Here, we have presented longitudinal HUI-3 and SSQ data for a small group of patients with COM receiving a bone conduction hearing implant. These data provide a unique view to the baseline self-assessed profile of these patients and presents utility values when rehabilitating with a bone conduction hearing implant, which have, to date, not been reported in the scientific literature.

### 4.1. Health Utilities Index Mark 3

The mean change in utility as measured by HUI-3 of 0.145 was clinically relevant. Pre-implant scores of 0.546 were in range with those previously reported for patients with an active or inactive middle ear disease [26]. Baseline scores indicated that patients with COM experience considerable burden in HRQoL, which is markedly higher than other ear diagnoses, including sensorineural hearing loss and dizziness [26]. This highlights the need for effective hearing rehabilitation in the COM population. Tympanoplasty and air conduction hearing aid provision have previously been shown to result in a statistically significant increase in HRQoL of 0.084 and 0.038 in patients with middle ear disease, respectively [26]. The change of 0.145 reported in our study is almost twice the utility gain compared to middle ear reconstructive surgery and almost four-times the utility gain provided by air conduction hearing aids. These data were supported by the complete case analysis and suggests that providing patients with a bone conduction hearing implant may be a more effective intervention compared to both surgical reconstructions that aim to restore hearing and air conduction hearing aids. This may be because patients with COM typically rate their hearing loss as a primary issue [27] and bone conduction hearing implants are uniquely suited to rehabilitate hearing in patients with COM, as they bypass the middle ear and leave the external ear unoccluded to offer stable hearing amplification that is independent of changes in middle ear status [28]. This assumption is supported by the hearing attribute scale in HUI-3, where patients reported a mean pre-intervention score of 0.858, demonstrating the impact their hearing loss has on the daily life. This score improved to 0.899 one year after patients received their bone conduction hearing implant, representing a clinically relevant improvement in hearing attribute. Statistically significant and clinically important improvements in HUI-3 hearing attribute are routinely observed as reported in other interventional studies investigating bone conduction devices in mixed indications [29,30]. Our results are particularly encouraging since they demonstrate that bone conduction implantation leads to a measurable increase in both hearing-related HRQoL and overall HRQoL through self-assessment in patients with COM. A small decrease in mean speech attribute score of 0.009 was captured between pre- and post-implantation, but this did not represent a clinically relevant change and is likely due to ceiling effects, since patients reported high scores for this attribute at pre-implantation and follow-up [31].

### 4.2. Speech, Spatial and Qualities of Hearing Scale-49

Since generic instruments may not be sensitive enough to capture specific aspects of health, such as hearing-related burden, in daily life, the HUI-3 questionnaire was supplemented with the disease-specific SSQ questionnaire to provide a more comprehensive overview of the hearing burden experienced by patients with COM in their daily lives. It is important to characterize the hearing burden more comprehensively as the HUI-3 hearing attribute score was particularly low in relation to other attribute scores. The hearing domain also suffers from an inherent ceiling effect that is most pronounced in the normal hearing situation (i.e., aided situation) [32]. Between baseline and post-implantation, patients reported a statistically significant and clinically relevant mean improvement in global SSQ score of 1.65. This change was mainly driven by a clinically relevant and statistically significant improvement in spatial domain score, although improvements in mean score were seen across all SSQ domain scores. Hearing handicap is potentially influenced by several dimensions of spatial hearing, impacting conversational competence, but all domains have some interdomain association [17]. Improvements in SSQ scores typically improve post-intervention in patients implanted with a hearing device [33], although it is less common for the largest improvement to be observed in the spatial hearing domain [33,34]. We can speculate that the large improvement in the spatial domain score in our study may be explained by the fact that patients with COM predominantly experience a mixed or conductive hearing loss and patients with single-sided deafness are unlikely to experience the same sound localization benefits as patients with purely conductive or mixed hearing losses, since binaural hearing will not be restored as sounds will be conducted to the functional contralateral cochlea [35]. Improvements in general HRQoL captured by HUI-3 were corroborated by the SSQ data.

### 4.3. Strengths and Limitations

The primary strength of this study is that it provides HRQoL data that are specific for patients with COM undergoing implantation with bone conduction hearing devices. The study also provides information on hearing disability faced by patients with COM in their daily lives via the SSQ. However, these data are derived from a single-arm study conducted on a small sample of patients, which limits the strength of evidence. There may also be bias from loss to follow-up, since reasons for missed follow-ups were not captured. However, as the effect size is independent of sample size, it is clear that the intervention resulted in an impactful change for patients, post-implantation. However, Cohen’s d may overestimate effect size due to bias of standard deviation in small samples, and as effect size is rarely reported in otology research it is difficult to put the magnitude of these changes into perspective. Studies implementing stronger study designs and more thorough reporting and that are less susceptible to bias, such as randomized controlled trials, investigating the influence that various hearing interventions have on patients with COM are therefore warranted to strengthen the current evidence base and measures of effect size. Additionally, as HRQoL and hearing disability are known to be differentially impacted by the degree and type of hearing loss [36], it will be important to capture the relationship between these factors and health outcomes in future studies. Furthermore, there is still an outstanding need for more sensitive generic QoL tools sensitive to hearing treatment effects that also consider different degrees of hearing loss and situations such as listening effort [37]. A tool such as this would bring great value to future assessments and would facilitate improved care for patients with hearing impairment in general.

## 5. Conclusions

Mean HUI-3 and SSQ scores improved for patients with COM who underwent bone conduction hearing device implantation. Bone conduction implantation was found to improve hearing and HRQoL for patients with COM, with or without cholesteatoma, resulting in a mean utility gain of 0.145 between pre-implantation and 12-month follow-up, post implantation. This information demonstrates the importance of providing appropriate hearing rehabilitation for individuals with COM to improve the influence their hearing deficit places on their daily life. Data also demonstrate the potential for implantable hearing devices in alleviating the public health burden of COM-related hearing loss.

## Figures and Tables

**Table 1:** Patient demographic and background data for the HUI-3 and SSQ cohorts at pre-implantation and follow-up.

	HUI-3	SSQ
Number of patients	10	6
Mean age in years (SD)	48.5 (15.0)	45.0 (16.6)
Sex: male; female	5; 5	4; 2
Pure tone average (500, 1000, 2000 and 4000 Hz) bone conduction threshold for implanted ear (dB) *	33.2	35.3
Country of residence		
Colombia	9	5
Poland	1	1

* bone conduction hearing threshold data missing for 1 patient.

**Table 2:** Mean (SD) HUI-3 attribute scores, utility score, and change in score for patients with COM, with or without cholesteatoma, at pre-implantation and at 12-month follow-up (*n* = 10).

	Pre-Implantation	Follow-Up	Change
Vision	0.978 (0.049)	0.986 (0.010)	0.008
Hearing	0.858 (0.152)	0.899 (0.097)	0.041
Speech	0.966 (0.047)	0.957 (0.103)	−0.009
Emotion	0.935 (0.063)	0.950 (0.058)	0.015
Pain	0.915 (0.133)	0.968 (0.040)	0.053
Ambulation	0.993 (0.022)	1.000 (0.000)	0.007
Dexterity	0.995 (0.016)	1.000 (0.000)	0.005
Cognition	0.958 (0.072)	0.970 (0.057)	0.012
Utility Score:	0.546 (0.277)	0.691 (0.262)	0.145

## Data Availability

Aggregated, anonymized study data will be made available upon reasonable request by contacting the corresponding author.
